# Does a continuous local anaesthetic pain treatment after immediate tissue expander reconstruction in breast carcinoma patients more efficiently reduce acute postoperative pain - a prospective randomised study

**DOI:** 10.1186/1477-7819-12-16

**Published:** 2014-01-16

**Authors:** Branka Strazisar, Nikola Besic, Uros Ahcan

**Affiliations:** 1 Department of Anaesthesiology, Institute of Oncology, Zaloska 2, Ljubljana SI-1000, Slovenia; 2 Department of Surgical Oncology, Institute of Oncology, Zaloska 2, Ljubljana SI-1000, Slovenia; 3 Department of Plastic Surgery and Burns, University Clinical Centre Ljubljana, Zaloska 7, Ljubljana SI-1000, Slovenia

**Keywords:** Breast carcinoma, Primary reconstruction with tissue expander, Pain treatment, Wound infusion of local anaesthetic, Elastomeric pump

## Abstract

**Background:**

Immediate breast reconstruction with an expander is a reasonable option for properly selected patients. After reconstruction, patients have severe postoperative pain, which responds poorly to opioids. Our aim was to evaluate if continuous wound infusion of a local anaesthetic into the surgical wound reduces postoperative pain, consumption of opioids and incidence of chronic pain compared to standard intravenous piritramide after primary breast reconstruction in breast carcinoma patients.

**Methods:**

Altogether, 60 patients were enrolled in our study; one half in the group with wound infusion of a local anaesthetic, and the other half in the standard (piritramide) group. Parameters measured included: pain intensity (visual analogue scale), drug requirements, alertness, hospitalisation, side-effects and late complications. A p-value of < 0.05 was considered statistically significant.

**Results:**

In the recovery room, the test group reported less acute pain at rest (*P* = 0.03) and at activity (*P* = 0.01), and on the day of the surgical procedure they reported less pain at activity (*P* = 0.003). Consumption of piritramide and metoclopramide was lower in this group (*P* < 0.0001), but their alertness after the surgical procedure was higher compared to the standard group (*P* < 0.001). After three months, the test group reported less chronic pain (*P* = 0.01).

**Conclusions:**

After primary tissue expander breast reconstruction, wound infusion of a local anaesthetic significantly reduces acute pain and enables reduced opioid consumption, resulting in less postoperative sedation and reduced need for antiemetic drugs. Wound infusion of a local anaesthetic reduces chronic pain.

## Background

Breast cancer is the most common cancer in women both in the developed and the developing countries [1]. Although systemic therapy and conservative surgery are recommended treatments, mastectomy is still the standard surgical procedure for the local treatment of all stages of breast cancer [2]. In the United States, one third of newly discovered breast cancer patients decide to undergo a mastectomy, mostly because of fear of recurrence. For those women who choose mastectomy as part of their approach to breast-cancer therapy or prevention, reconstruction may be offered as an option [3]. In Breast Center Humanitas in Milan, breast reconstruction after mastectomy was performed in 82% of patients in 2010 [4].

As a reconstruction method, an expander or an implant provides a reasonable option in properly selected patients. Patients with small, minimally ptotic breasts are good candidates. The advantages of this method are simple operation, no morbidity at a distant donor site, use of tissue of similar colour, texture and sensation, reduced operating time, and faster postoperative recovery [5]. Although breast reconstruction with a tissue expander is simple, it causes severe postoperative pain that may respond poorly to opioids [6]. Severe postoperative pain after breast implant insertion is well-known from breast augmentation studies [7]. Pain relief was poor despite the progressive increase in the opioid dose and presence of adverse effects of opioids [6].

Use of local anaesthetics is one of the possible methods for the treatment of acute postoperative pain. To our knowledge, only three studies have been published on the use of local anaesthetics for the treatment of acute postoperative pain after breast reconstruction [8-10]. Use of local anaesthetics administered into the surgical wound has been shown to be an effective treatment for postoperative pain control and decreased opioid consumption. However, only a small number of patients were included in these studies. Only one of the studies was prospective and randomized [8] but it was performed on patients with delayed breast reconstruction after mastectomy. We failed to find any prospective randomized trial on the use of local anaesthetics for the treatment of postoperative pain after primary breast reconstruction with a tissue expander.

We decided to conduct study because of the good results obtained in previous studies on axillary lymph node dissection and mastectomy due to breast carcinoma [11-14]. Levobupivacaine and ropivacaine are long-lasting and very effective local anaesthetics with a favourable toxic profile [15]. Levobupivacaine was chosen for use in our patients because it was available on our market at the time of our study, while ropivacaine was not. The duration of action of local anaesthetic is proportional to the length of time that it is in contact with the nerve [16]. Pacik found that a continuous flow as well as intermittent bolus anaesthesia was effective in controlling postoperative pain in augmentation mammoplasty [17]. But according to Talbot *et al*., a bolus application of local anaesthetic was not a successful pain treatment after mastectomy [18], so we decided to use a continuous infusion of local anaesthetic. The aim of our prospective randomized study was to evaluate the effectiveness of a local anaesthetic administered into the surgical wound as a continuous infusion after immediate breast reconstruction with a tissue expander. Our hypothesis was that local anaesthetics are more effective than standard intravenous analgesics for pain relief. We also wanted to determine that continuous infusion of the local anaesthetic enables lower consumption of opioids, reducing the need for antiemetic drugs and sedation compared to standard opioid-based analgesia.

## Methods

Altogether, 60 breast carcinoma patients were enrolled in the study protocol between January 2011 and May 2012: 72 patients who were eligible for inclusion were offered the opportunity to participate in our study during the recruitment period; 12 of them declined participation, and the remaining 60 were included in the study. Patients listed for mastectomy and primary reconstruction with a tissue expander because of the presence of breast carcinoma or for prophylactic mastectomy, were randomized during the routine preoperative anaesthetic assessment. Anaesthesia for the procedure was the same for all study subjects. We used the same protocol as already reported for patients with axillary dissection [14]. The first study arm was treated with continuous local analgesia, and the other (control) arm received standard intravenous analgesia. Allergies to local anaesthetics and chronic pre-operative opioid consumption were the criteria to exclude patients from our study.

After informed consent was obtained the randomization was made by research nurses from the Clinical Research Unit of the Institute of Oncology Ljubljana. The research nurse performed randomization using random numbers generated by a computer and then informed the principal investigator about the treatment allocation of the patient.

Surgical procedures were performed by ten experienced oncology surgeons and ten plastic surgeons. Sentinel lymph node biopsy was performed in 55 patients. In one patient with positive imprint cytology of the sentinel lymph node, axillary lymphadenectomy was performed immediately.

The study was reviewed by the The National Medical Ethics Committee of the Republic of Slovenia and performed in accordance with the ethical standards laid down in the appropriate version of the 1964 Declaration of Helsinki. Our study was approved by the Institutional Review Board of the Institute of Oncology Ljubljana and conducted with the understanding and consent of all subjects involved. Study was entirely financed by the Institute of Oncology as a part of public service.

### Test group of patients

Before wound closure, a fenestrated wound catheter was placed by a plastic surgeon into the wound cavity under the musculus pectoralis major and upon the entire length over the upper side of the wound. The wound catheter was fenestrated along 15 cm in the distal part. Nine patients undergoing bilateral reconstruction received a catheter into the wound on both sides. A bolus of 15 mL of 0.25% levobupivacaine was injected into the wound through the catheter immediately after wound closure. Surgical drains and the fenestrated catheter were clamped for 5 minutes after the administration of levobupivacaine to enable its absorption. After the administration of the bolus, the elastomeric pump was connected to the wound catheter. Clamps on the surgical drains were released after 5 minutes, and the clamp on the fenestrated catheter was released after 20 minutes, enabling a continuous infusion of 0.25% levobupivacaine. The elastomeric pump contained 100 mL of 0.25% levobupivacaine, and the flow rate was 2 mL per h. The catheter was removed after 50 h.

### Control group of patients

The control patients, that is, those who received the standard intravenous piritramide treatment, were on a continuous intravenous infusion with piritramide (30 mg), metoclopramide (20 mg) and metamizole (2.5 g) in 100 mL of 0.9% sodium chloride (3 mL/h - 6 mL/h) until the next morning. The nursing staff were instructed to maintain the lowest drip rate of infusion, which relieved the pain. Our experience is that after the first postoperative day the severity of pain decreases and becomes bearable. In order to avoid the side effects of opioids the patients from our control group were treated with continuous intravenous infusion only during the first 24 h as is the standard of care in our department.

### Both groups of patients

Patient-controlled analgesia was not used in our patients. A rescue dose of analgesics was administered by nursing stuff on the demand of a patient. Whenever needed, patients from both groups could get an intravenous bolus of a rescue analgesic (3 mg of piritramide) and/or an antiemetic drug. In the case of nausea or vomiting, the patient received a bolus of an antiemetic drug, the first one being metoclopramide. If no relief was achieved, 1 mg of granisetron or, with persistent nausea 1.25 mg of droperidol, was administered intravenously.

From the first postoperative day, all patients received analgesics in the form of tablets. They were administered one tablet of 100 mg of diclofenac and a combination of paracetamol 2,600 mg per day and tramadol 300 mg per day. The consumption of drugs (analgesia infusion, intravenous drugs and tablets) during hospitalisation was registered.

### Pain measurement

Data about pain (visual analogue scale (VAS) score) were collected by nursing staff, that is, by an independent observer. Pain was measured using the standard VAS score, ranging from 0 to 10. The first measurement was made in the recovery room. Pain was also measured 3, 6 and 9 h after the surgical procedure. Thereafter, pain was measured every 8 h over the next 4 days. All VAS measurements were performed twice, at rest and on activity of an upper extremity. Median VAS score was calculated on the day of surgery and on the first postoperative day from all eight and six measurements, respectively.

Three months after the surgical procedure, that is, before the tissue expander was replaced by an implant, the patients were asked about pain in the postoperative area or upper extremities. Chronic pain after 3 months was defined as pain that bothered patients during normal daytime activities or during rest or sleep. In the majority of patients with persistent pain it occurred during movement of an upper extremity or in a specific position of the extremity. Pain was permanently present when at rest in only a minority of patients. Six hours after the surgical procedure, we measured patients' alertness using the observer’s assessment of alertness/sedation (OAA/S) scale [19] in a composite way, as we did in our previous study [14].

After the surgical procedure, the patients received adjuvant therapy according to the tumour stage and guidelines. Adjuvant chemotherapy, external irradiation and/or hormone therapy was used in 38%, 20% and/or 60% of the patients, respectively. The occurrence of complications (inflammation, haematoma and other) was recorded.

### Statistical analysis

In order to estimate the probability (power) to reject the null hypothesis, PS: Power and Sample Size Calculations Software (Version 3.0, January 2009) was used. Prior data indicated that the failure rate among controls was 0.5. If the true failure rate for experimental subjects is 0.15, 27 experimental subjects and 27 control subjects are needed to be able to reject the null hypothesis that the failure rates for experimental and control subjects are equal, with probability (power) of 0.8. The type-I error probability associated with this test of the null hypothesis is 0.05.

The Student *t*-test or the Mann–Whitney *U*-test was used according to data distribution. The association between categorical variables was tested by the chi-square test or Fisher's exact test, as appropriate. All comparisons were two-sided and a *P*-value <0.05 was considered statistically significant. The statistical packages PASW 18 (SPSS Inc., Chicago, IL, USA) and R 2.11.1 (R Foundation for Statistical Computing, Vienna, Austria) were used for the analysis.

## Results

The mean age of patients was 47.8 years (range 25 to 64 y), height was 166 cm (range 153 to 178 cm), weight was 61.7 kg (range 45 to 85 kg), and the body mass index (BMI) was 22.4 (range 17 to 31.2). There were no significant differences between the study groups in BMI, American Society of Anesthesiology (ASA) score, comorbidities (Table 1), type and length of surgical procedure, extent of lymph node dissection, adjuvant therapy, complications, or hospital stay (Table 2).

**Table 1:** Patient characteristics

**Characteristic**	**Sub-group**	**Local anaesthetic group**	**Standard group**	***P*-value**
Number of patients		30	30	-
Age, years, mean		47.6	48.0	0.88
Height, m, mean		1.66	1.66	0.78
Weight, kg, mean		60.9	62.5	0.56
Body mass index, kg/m^2^, mean		22.2	22.6	0.61
ASA score	1	10	15	0. 44
	2	19	14	
	3	1	1	
Associated diseases, n		15	14	1.0
Smoker, n		9	10	0.93
Diabetes mellitus, n		0	0	-
Fibromyalgia, n		0	0	-
Rheumatoid arthritis, n		0	0	-
Depression, n		3	1	0.61
Mastectomy side	Left n	7	15	0.10
	Right n	15	10	
	Both n	8	5	
Tumour diameter, cm, mean (range)		1.2 (0 to 5)	1.3 (0 to 6.5)	0.76
Histology (n = 60)	Invasive ductal carcinoma, n	22	17	0.56
	Invasive lobular carcinoma, n	2	2	
	Ductal *in situ*, n	1	2	
	Without carcinoma, n	5	9	
Grade (n = 58)	Grade 0, n	5	9	0.66
	Grade I, n	2	1	
	Grade II, n	11	9	
	Grade III, n	11	10	
Metastatic lymph nodes, median (range)		0 (0 to 21)	0 (0 to 18)	0.98
Resected lymph nodes, median (range)		2 (0 to 33)	3 (0 to 27)	0.97
Hormone receptor-positive n		18	20	0.79
HER-2-positive n		5	7	0.75

ASA, American Society of Anesthesiology; HER-2, human epidermal growth factor receptor-2; n, number of patients.

**Table 2:** Treatment of patients, adjuvant therapy, hospital stay and complications

	**Sub-group**	**Local anaesthetic group**	**Standard group**	***P*-value**
Breast surgical procedure (n = 73)	Mastectomy; one-sided	23	24	0.58
	Mastectomy; two-sided	7	6	
Lymph node surgical procedure	Axillary lymphadenectomy	6	5	1.00
	Sentinel lymph node biopsy; one sided	21	22	1.00
	Sentinel lymph node biopsy; two-sided	3	3	
	Without sentinel	6	5	
Reconstruction	Expander; one-sided	21	20	1.00
	Expander; two-sided	9	10	
Median duration of surgical procedure, minutes		140	120	0.62
Neoadjuvant chemotherapy	Yes	4	3	1.00
	No	26	27	
Postoperative chemotherapy	Yes	13	10	0.60
	No	17	20	
Postoperative radiotherapy	Yes	9	3	0.11
	No	21	27	
Hormone therapy	Yes	17	19	0.79
	No	13	11	
Hospitalisation, days		5.2	5.4	0.61
Haematoma		2	1	1.00
Revision		1	1	1.00
Inflammation		1	3	0.61

Values are numbers of patients unless stated otherwise.

### Acute pain

Data on postoperative pain are presented in Table 3.

**Table 3:** Pain in the local anaesthetic group and the standard group of patients

**Pain assessment time point**		**Local anaesthetic group**	**Standard group**	***P*-value**
In recovery room	At rest	3.0	4.0	0.03
	On activity	3.0	5.0	0.01
3 h after operation	At rest	1.0	1.5	0.45
	On activity	3.0	5.0	0.04
6 h after operation	at rest	2.0	2.0	0.58
	On activity	3.0	5.0	0.009
9 h after operation	At rest	1.0	2.0	0.28
	On activity	4.0	5.0	0.01
Day of surgery	At rest	1.9	2.1	0.23
	On activity	3.8	4.8	0.003
First postoperative day	At rest	1.5	1.7	0.69
	On activity	4.0	3.7	0.96
3 months postoperative (number of patients still having pain)		5	15	0.01

Data are presented as median visual analogue scale (VAS) score unless stated otherwise.

### Opioid consumption

Consumption of piritramide during the first 24 h after the surgical procedure was lower in the test group compared to the control group (*P* <0.0001) (Table 4). Alertness, as measured 6 h after the surgical procedure, was higher in the test group compared to the control group (*P* <0.001).

**Table 4:** Mean consumption of drugs, and alertness, in the local anaesthetic group and the control group of patients

**Pain**	**Local anaesthetic group**	**Standard group**	***P*-value**
Opioid (piritramide) consumption during the first 24 h, mg, mean	9.8	29.4	<0.0001
Metamizole consumption during the first 24 h, g, mean	2.8	4.5	<0.0001
Metoclopramide consumption during the first 24 h, mg, mean	11.0	24.3	<0.0001
Tramadol/paracetamol consumption during the first four days, number of tablets, mean	16.8	18.8	0.46
Diclofenac consumption during the first four days, mg, mean	324.1	340.4	0.57
Alertness OAA/S score 6 h after surgery, mean	5.0	4.4	<0.001

OAA/S - observer's assessment of alertness/sedation.

### Nausea

Patients in the test group reported less nausea than patients in the standard group. Consumption of metoclopramide during the first 24 h after the surgical procedure was also lower in the test group compared to the standard group (*P* <0.0001).

### Complications

No local signs of an infection were observed in the area where the wound catheter was inserted. All microbiological samples taken were negative. There were no significant differences in the complications following the surgical procedure between the two groups. Altogether, two patients (3.3%) underwent another surgical procedure because of haematoma, one from the test group and one from the standard group. Inflammation after the surgical procedure occurred in four patients, one in the test group and three in the standard group. After antibiotic therapy, all patients recovered, and the tissue expander stayed in place.

### Hospital stay

The average postoperative hospital stay was 5.3 days. There was no significant difference in the duration of the hospital stay between the two groups.

### Unilateral and bilateral tissue expander

In order to collect a sufficient number of patients all cases with a unilateral or bilateral tissue expander were included in our study. As a result of randomization the number of the patients with a bilateral and unilateral tissue expander reconstruction in the local anaesthetic and control group was nine and ten, respectively. Patients with bilateral tissue expander reconstruction received 2 mL/hour of 0.25% levobupivacaine on each side. Thus, the total daily amount of levobupivacaine was 240 mg, which is within the recommended range and does not exceed the maximum tolerated dose. Patients from the control group with a bilateral reconstruction had more severe pain than patients with a unilateral reconstruction, whereas pain in the local anaesthetic group was not different after a bilateral or unilateral reconstruction. In the control group the mean VAS score in the recovery room after a bilateral and unilateral reconstruction was 6.45 and 4.09, whereas on the day of surgery it was 5.36 and 3.13, respectively (*P* <0.05).

### Late complications (three months after primary reconstruction)

Three months after primary reconstruction, the patients completed a questionnaire on pain. In the test and the control groups of patients, pain was reported in 16.7% and 50% (*P* = 0.01), respectively. Unilateral lymphoedema of the arm was present in two patients, one from the control group and one from the test group. The patient with oedema from the test group underwent a dissection of the axilla; the patient from the control group underwent a bilateral sentinel lymph node biopsy.

## Discussion

Ideally the study should have been placebo double-blinded to eliminate all biases. However, the cost of the elastomeric pump and wound catheter was 175 euro, so we were not able to perform a double-blind study. However, the cost of an elastomeric pump and wound catheter was not the only reason that precluded us from performing such a study. We had also ethical reasons for not carrying out a placebo-controlled study. Namely, a reconstruction with a tissue expander is a very painful procedure for the majority of patients, so we felt that it is not ethical to have a control group treated by placebo only. Instead, we offered our control group of patients the best available treatment, that is, intravenous analgesia. Based on the good results of our preliminary data on the use of continuous local anaesthetics for treatment of pain, our patients were willing to participate in our study because they were offered state-of-the-art pain treatment versus potentially even more effective pain treatment.

Sub-pectoral breast augmentation and breast reconstruction with an expander or an implant are associated with considerable pain in the early postoperative period [6,9,10,20-24]. Legeby *et al*. [6] found that pain in the first 3 h after immediate breast reconstruction with a tissue expander was more severe than pain after mastectomy or axillary dissection. The mean VAS value for pain in the first 3 h after breast reconstruction was 4.9 and 5.0, if axillary dissection was also performed, whereas the values were 2.1 for mastectomy and 3.0 for mastectomy with axillary dissection [6]. Similarly, after tissue-expander implantation, our standard analgesia group of patients also suffered pain in the recovery room, which was by about 3.0 VAS units larger than in patients who underwent breast surgery with axillary dissection without reconstruction, as reported by Strazisar *et al*. [14]. The mean VAS score after immediate reconstruction was 4.9 in the standard analgesia group, whereas it was only 3.4 in the local anaesthetic group. In our previous study, the mean VAS score in recovery after breast cancer surgery with axillary dissection was 2.0 in the standard control group and 0.5 in the local anaesthetic group [14]. In both our studies, the local anaesthetic reduced pain by 1.5 VAS units more than standard treatment alone.

The most positive result of our study is the observation that 77% of patients treated with local anaesthetics did not experience severe pain. To our knowledge, there are no reports in the literature about similarly effective early postoperative analgesia after the implantation of a breast-tissue expander. Legeby *et al*. reported that even 48% of patients treated with local anaesthetics experienced unacceptably severe pain (VAS 4.7 to 10.0) in the early postoperative period [6]. Legeby *et al*. used an epidural catheter with only a few holes at its tip and instilled local anaesthetic every 3 h. They probably did not establish an equal distribution of the local anaesthetic along the entire length of the muscle and the wound. On the other hand, we used quality wound-catheters that were perforated 15 cm at the distal end, which enabled a better distribution of the local anaesthetic. Furthermore, we used an elastomeric pump, which enabled a continuous infusion of the local anaesthetic. That is probably the reason why only seven patients (23.1%) in the test group reported pain above a VAS score of 5.0, whereas in the control group, such pain levels were reported by thirteen patients (42.9%).

Legeby *et al*. found that the use of opioids was about three times higher after the implantation of a tissue expander compared to other breast surgical procedures [6]. After the tissue-expander implantation with or without axillary dissection, the consumption of opioids was 12.8 mg and 11.8 mg, whereas after mastectomy or mastectomy with axillary dissection, the mean consumption of opioids was 3.2 mg and 5.5 mg, respectively [6]. Our patients with standard analgesia after the tissue-expander implantation needed 5.6 times more opioids than patients who underwent axillary dissection alone [14]. Local anaesthetics can reduce consumption of opioids after mastectomy and axillary dissection [11,13,14] and also after breast augmentation with implants [7]. Our study shows that the use of continuous local anaesthesia can effectively reduce opioid consumption. Our patients treated with a local anaesthetic needed about three times less opioids than the standard analgesia group. Subsequently, patients from the local anaesthesia group experienced nausea less often, thus they had a lower consumption of metoclopramide than the standard analgesia group. Furthermore, the use of local anaesthetics resulted in less sedated and more alert patients in the recovery room and 6 h after the operation. The complication rate did not differ between the two groups of patients. This is in agreement with the reports of other authors who used local anaesthetics [11-13,18,21,25-30]. Some authors report that local anaesthetics appear to reduce the incidence of inflammation [31-33].

There was no difference in pain on the first postoperative day between the study and control group of patients. It means that local anaesthesia was no longer as effective as it was on the day of surgery. A possible explanation is that muscle overstretching caused by a tissue expander is no longer as painful as it was. Other explanations are that in some patients the position of the wound catheter might have changed during movement or that there was a different perfusion region in the erect, prone or supine position, or that continuous infusion was flawed because of the wound drainage. Unfortunately, in our study we did not test a rescue bolus-injection of levobupivacaine in the case of severe pain. However, we have good experience with application of a rescue bolus of levobupivacaine in patients with neuropathic pain after axillary lymphadenectomy. Many times we have observed that injection of a bolus of 15 mL of 0.25% levobupivacaine into a drain and clamping of wound drainage inhibited neuropathic pain for a couple of hours.

Memory of severe postoperative pain is the most important risk factor for the development of chronic pain [34]. Patients who were treated with patient-controlled analgesia had three times less chronic pain than the traditionally nurse-administered patients [6]. The results of our study support this observation. Only 17% of patients treated with a local anaesthetic experienced chronic pain. This is significantly less than in the standard analgesia group of patients, of whom 50% suffered chronic pain. The proportion of patients treated with axillary lymphadenectomy, chemotherapy, hormone therapy and/or irradiation was similar in both study arms. Hence, we think that inadequately treated acute pain had an impact on the frequency of chronic pain in the standard analgesia group.

## Conclusions

Our current prospective randomized study shows that a continuous local anaesthetic infusion with the use of analgesic catheters inserted into the surgical wound after primary breast reconstruction with a tissue expander reduces acute pain immediately after the surgical procedure and also on the operative day. Because of that, it represents an effective postoperative pain treatment without side effects. It enables lower opioid consumption in the first 24 h after the surgical procedure, higher alertness and less nausea. We observed that patients treated with a local anaesthetic experienced a lower frequency of chronic pain compared to patients treated with standard analgesia.

## Abbreviations

BMI: body mass index; ASA: American Society of Anesthesiology; VAS: visual analogue scale; OAA/S: observer’s assessment of alertness/sedation.

## Competing interests

The authors declare that they have no competing interests.

## Authors’ contributions

BS - made the study design, carried out the study and drafted the manuscript, NB - co author of the study design and co drafted the manuscript, UA - consultant and co drafted the manuscript in the part of Synopsis. All authors read and approved the final manuscript.
